# Finding the Right Balance: A Social Norms Intervention to Reduce Heavy Drinking in University Students

**DOI:** 10.3389/fpubh.2021.653435

**Published:** 2021-06-10

**Authors:** Christine Wolter, Tino Lesener, Tobias Alexander Thomas, Alicia-Carolin Hentschel, Burkhard Gusy

**Affiliations:** ^1^Division of Public Health: Prevention and Psychosocial Health Research, Department of Education and Psychology, Freie Universität Berlin, Berlin, Germany; ^2^Institute for Experimental Psychology, Faculty of Mathematics and Natural Sciences, Heinrich-Heine-Universität, Düsseldorf, Germany; ^3^Department of Psychosomatic Medicine and Psychotherapy, Hannover Medical School, Hanover, Germany

**Keywords:** social norms intervention, prevention of alcohol misuse, University students, alcohol intervention, heavy drinking

## Abstract

**Introduction:** Heavy alcohol consumption constitutes a major health risk among University students. Social relationships with peers strongly affect University students' perception of the drinking behavior of others, which in turn plays a crucial role in determining their own alcohol intake. University students tend to overestimate their peers' alcohol consumption – a belief that is associated with an increase in an individual's own consumption. Therefore, we implemented a social norms intervention with personalized normative feedback at a major University in Germany to reduce and prevent excessive drinking among University students.

**Methods:** Our intervention was part of a regular health monitoring survey. We invited all enrolled University students to take part in this survey on two occasions. A total of 862 University students completed the questionnaire, 563 (65.3%) of which received e-mail-based feedback upon request concerning their peers' and their own alcohol consumption. For the intervention group (*n* = 190) as well as the control group (no feedback requested; *n* = 101), we included only University students in the evaluation who overestimated their peers' alcohol use and indicated above average consumption of the peers. We applied analyses of variance to assess intervention effects with regard to the correction of overestimated group norms as well as University students' drinking behavior.

**Results:** Within the intervention group, we observed a significantly larger reduction of the previously overestimated behavioral norms compared to the control group (*p* < 0.001; $\eta_{\text{p}}^{2}$ = 0.06). With regard to behavioral outcomes the intervention group showed a significantly larger reduction in the AUDIT-C score (*p* = 0.020; $\eta_{\text{p}}^{2}$ = 0.03).

**Discussion:** Our study confirms previous research whereupon personalized, gender-specific and selective normative feedback is effective for alcohol prevention among University students. However, University students still overestimated their peers' alcohol intake after the intervention. Furthermore, we did not reach high-risk groups (University students with the highest alcohol intake) since no feedback was requested. Future studies should address factors influencing the impact of the intervention and reachability of selective groups.

## Introduction

Harmful use of alcohol causes about 3 million deaths each year and more than 130 million disability-adjusted life years (1). The mortality caused by alcohol is higher than that caused by diseases such as tuberculosis, HIV/AIDS, or diabetes (1). Constant alcohol use causes social impairments and increases the risk for various serious diseases, like alcoholic cirrhosis, tumors and cancer, as well as premature mortality (2, 3). Especially younger adults are disproportionally affected by alcohol: More than 13% of all deaths between 20 and 39 are attributed to harmful use of alcohol (1). The highest prevalence rates of alcohol use disorders are in high-income countries, especially in Europe and in the US. Heavy alcohol consumption is also highly prevalent among young adults in Germany: 42% of men and 33% of women between 18 and 29 display heavy consumption patterns (4). Research suggests that—within this age group—University students tend to drink even more (5, 6) and also more frequently (5) than their non-University peers. Alcohol consumption is widespread among German University students: On average, two thirds of the University students drink alcohol at least twice a month. Nearly one third of the University students report binge drinking at least once a month, and more than 40% show problematic drinking behavior, i.e., an AUDIT-C sum of at least 3 in women and at least 4 in men (7).

Consequently, and due to these high prevalence rates of heavy alcohol consumption and negative outcomes concerning health, social life and society in general there is a particularly high urgency to address the alcohol consumption of University students with proper interventions in order to prevent early-onsets of heavy drinking behavior. These interventions need to simultaneously reduce the harmful use of alcohol and strengthen responsible and low-risk handling of alcohol.

### How Do Peers Affect University Students' Alcohol Consumption?

Heavy drinking behavior is affected by intrapersonal and interpersonal social and normative factors (8). Among University students, social relationships with peers play a crucial role for their drinking behavior (9). Accordingly, University students report drinking motives such as social enhancement, enjoyment, and socialization (10, 11). As a pioneer of social conformity theory, Asch (12) showed the impact of social pressure on individual behavior more than six decades ago. The perception of others' behavior—especially peers—affects University students' alcohol consumption through social norms as social influences (13). Moreover, University students adopt the drinking patterns they perceive in their peers. To prevent heavy alcohol consumption, interventions could therefore address social influences through social norms.

### Why Do University Students Overestimate Their Peers' Alcohol Consumption?

The perception of behavior is biased and therefore often differs from the behavior actually shown (14). This discrepancy is particularly noticeable in alcohol consumption (15, 16). The core of such misperceptions usually lies in an overestimation of others' risk behaviors and an underestimation of health-promoting behaviors (13). Several studies at US (17–19) as well as European universities (20–22) showed that University students systematically overestimate their peers' alcohol consumption. Thus, the fact—and behavioral norm—that the majority of University students use alcohol responsibly is disguised by individual misperceptions (19). Within literature on the causes for this overestimation, there are several explanations: First, it is argued that overestimation is due to the size of the peer group. As there is little/no information about unknown persons within the peer group there is a lack of information on their alcohol use and thus overestimation occurs (23, 24). Second, it was postulated that among the peer group only close peers (e.g., significant others) are used for estimating alcohol use. Thus, overestimation is a product of “underinclusion” as significant others are only a part of the peer group. However, this approach was disproved when being tested empirically (25). Third, it is proposed that this overestimation is due to a cognitive bias resulting in better memory and attention for more extreme behavioral patterns. Furthermore, this more extreme behavior is regarded as transsituationally consistent (26). Finally, overestimation of peers' alcohol use is found to be moderated by time (e.g., “seasonal effects”) as well by own alcohol intake as well as by a loss of self-control (25). This may eventually result in risky drinking behavior since misperceived behavioral norms may encourage individuals to adapt their alcohol intake to what they (mis-)perceive in their peers (13). The *social norms intervention* and *personalized normative feedback* approaches promisingly attempt to break this cycle [i.e., “you (unintentionally) drink more because you expect higher intake levels of your own based on peers' evaluation”; (13)].

### How Could We Change the Overestimation?

The *social norms intervention* (SNI) is a health-promoting intervention that aims to correct misperceptions by providing information about the behavioral norm in a population in order to support more health-conscious decision-making processes (27). This intervention approach assumes that correcting the misperceived behavioral norm by replacing it with the actual norm reduces the individual's pressure with regard to the mistakenly overestimated peer consumption (27). The SNI is based on two basic assumptions: (1) accurate information about the beliefs and behaviors of relevant others is not always known and salient, (2) providing the behavioral norm may change the understanding of group norms and one's own position within the group (28). The SNI differs from traditional behavioral change approaches. It focuses on indirect methods of persuasion by presenting information about (health-conscious) behavioral norms that already exist within a group (29). SNIs do not aim to change the behavioral norms but to correct misperceptions of that behavioral norm (13). The given information is a positive statement showing that responsible and moderate behavior is the behavioral norm, and that the group majority acts and thinks health-consciously (30).

With *personalized normative feedback* (PNF), each person receives individual, personal feedback, e.g., on their own as well as their peers' alcohol consumption (28). For this purpose, a mode of communication is chosen that allows for this kind of feedback, like face-to-face conversations in counseling (31), e-mails (32) or web-based messages via a personal link to a website (33).

### What Is the Current Evidence?

Intervention studies (34–38) and systematic reviews (27, 39, 40) demonstrate small to medium effects of SNIs on various alcohol-related outcomes such as drinking quantity, frequency and risky drinking. However, these results often do not persist in the long term. Neighbors et al. (37) showed significant short-term effects of their intervention (PNF, specifically) but no long-term effects on the individual estimation of the behavioral norms, alcohol frequency and quantity. Foxcroft et al. (41) reported inconsistent results in their meta-analytical review: While some studies found significant short- and long-term effects of SNIs and PNFs on alcohol quantity and binge drinking, other studies did not [see also (42)]. Additionally, the overall effect size was very small. However, Dotson et al. (43) declared even small effects as clinically relevant from a public health perspective. Referring to the “prevention paradox” even small improvements at the individual level might achieve large health gains at the population level. In their review, PNF was proven an effective stand-alone approach for reducing college student drinking (43). Moreover, some recent studies were able to obtain medium- and long-term effects of PNFs on drinking frequency after 3 months (44) and drinking prevalence after 6 months (45).

PNF has proven to be effective in correcting misperceived drinking behavioral norms (37, 46, 47). This modification of misperceived behavioral norms has been found to mediate the relationship between PNF and behavioral outcomes with regard to reduced drinking levels (38, 47–49).

### How Can SNIs Be Improved?

Several aspects might improve the efficacy of SNIs, such as the frequency, the reference group, or the selection of the intervention group. Samson and Tanner-Smith (50) meta-analytically showed that even one single session of PNF might have the same positive impact on alcohol consumption in the short- and medium-term as motivational enhancement therapy, motivational interviewing, or even more elaborated techniques. Most PNF interventions refer to “typical University students” as the normative peer group (43, 51). However, recent research highlights the importance of personal significance of the reference group to the individual. Close reference persons such as friends or peers, as well as factors like specificity (e.g., gender specificity) seem to have a greater impact on individual alcohol consumption than less close or specific reference groups such as “typical University students” (35, 52–54). Furthermore, Haug et al. (55) argue for selective SNI for persons who consume more alcohol than the average, as the intervention was more effective in studies that pre-selected persons with problematic alcohol use [see also (48)].

In summary, research shows that email-based PNF for alcohol prevention and reduction of alcohol consumption is effective among University students. Interventions seem to be most effective when they are personalized, gender-specific, and targeted at University students who drink more alcohol than the average of their peers.

### What Should We Do?

While interventions based on social norms are popular and widely applied at US universities, interventions that address University students in Europe and especially Germany are rare (41). SNIs have established themselves in the US as a meaningful way to reduce alcohol consumption. In order to popularize this type of intervention in Germany, the effects of an SNI were tested in this study.

Based on the existing evidence, we expect the effects of our PNF to be two-fold: First, we expect a correction of University students' misperceived behavioral norms with regard to their peers' alcohol consumption. Second, we expect a reduction of alcohol intake on the behavioral level.

The intervention aims to specifically address students who overestimate their peers' alcohol consumption and consume more than the average of their peers. Consequently, our hypotheses are:

*Hypothesis 1: The intervention (personalized normative feedback) corrects misperceived behavioral norms in University students who overestimate the alcohol intake of their peers and consume more alcohol than the average of their peers at 12 weeks after the intervention*.

*Hypothesis 2: The intervention (personalized normative feedback) reduces levels of alcohol intake on the behavioral level in University students who overestimate the alcohol intake of their peers and consume more alcohol than the average of their peers at 12 weeks after the intervention*.

## Materials and Methods

### Study Procedure and Sample

**Figure 1:**
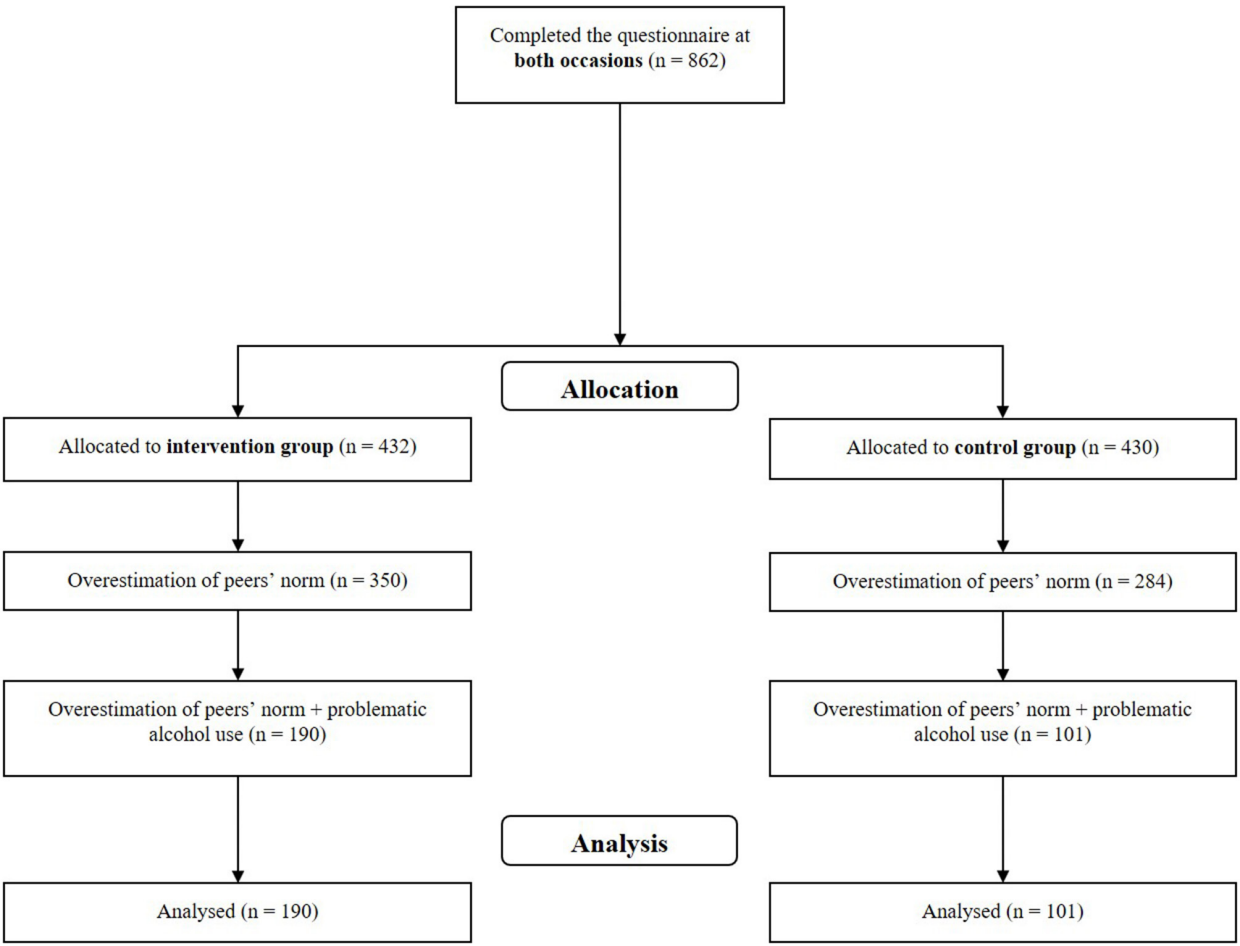
Flowchart of our study.

Our intervention was part of a general health monitoring survey at a major University in Germany. The survey covered University students' perception of study characteristics, health outcomes as well as their health behavior. All University students enrolled at the University were invited to take part in the survey. The survey was conducted twice (January/February and June/July 2019), with a total of 862 University students (mean age: 24 years; see Figure 1) participating. Two months (6–10 weeks) after the first survey, 563 (65.3%) University students received feedback upon request concerning their own and their peers' alcohol consumption. In the second survey, 432 (76.7%) University students of those who had received feedback indicated that they had actually read the feedback. University Students who had not taken part in the intervention (no feedback requested or not read the feedback) were assigned to the control group. The gender ratio (♀:♂) was 3:1 in each group. All subjects answered the questions on alcohol consumption on both occasions.

Within our a-priori defined subgroup analysis, we included only University students who overestimated their peers' alcohol consumption and who had indicated above average own consumption compared to the median of the peers' consumption. We refer to above average alcohol consumption as “heavy drinking.” No randomized assignment to the groups was possible. Consequently, 190 students fulfilling these criteria and were assigned to the intervention group and 101 students that also met these criteria to the control group. Those who did not fulfill inclusion criteria but wanted feedback were also given feedback. Thus, every University student requesting feedback received one.

### Measures

To assess University students' individual alcohol intake, we used the Alcohol Use Disorders Identification Test Consumption [AUDIT-C; (56)]. The AUDIT-C consists of three items of the original 10-item AUDIT. Each question (e.g., “How often do you drink an alcoholic beverage e.g., one glass of wine, beer, cocktail, schnapps or liqueur?”) is scored from 0 (e.g., “never”) to 4 (e.g., “6 or more times a week”) points, resulting in a score from 0 to 12. An AUDIT-C score of 0 means that participants never drink alcohol.

We utilized frequency-quantity-indices, combined values containing information about both frequency and quantity of alcohol use, within our analyses. These indices were also applied to assess the department- and gender-specific individually estimated group norm. We first asked University students to evaluate their peers' alcohol intake with regard to frequency (“How often does the majority of all female students in your department drink an alcoholic beverage e.g., one glass of wine, beer, cocktail, schnapps or liqueur?”) and quantity [“How many alcoholic beverages does the majority of all female students in your department usually drink per drinking occasion? One alcoholic beverage (standard drink) is a small bottle of beer (0,33l), a small glass of wine or sparkling wine (0,125l) or a double schnapps (4cl)”]. The items were adopted from AUDIT-C for estimation of peers' alcohol frequency and quantity. We then multiplied frequency and quantity and thus obtained the frequency-quantity index for the individually estimated group norm.

We computed another frequency-quantity index indicating the behavioral norm (i.e., median of peers' alcohol frequency multiplied with median of peers' alcohol quantity) and compared them by using a difference value (individually estimated group norm – behavioral norm) for the two frequency-quantity indices. By using the term overestimation, we refer to any difference value > 0 (meaning the individually estimated group norm is larger than the behavioral norm). We utilized frequency-quantity-indices, combined values containing information about both frequency and quantity of alcohol use, within our analyses. These indices were also applied to assess the department- and gender-specific individually estimated group norm. We first asked University students to evaluate their peers' alcohol intake with regard to frequency (“How often does the majority of all female students in your department drink an alcoholic beverage e.g., one glass of wine, beer, cocktail, schnapps or liqueur?”) and quantity [“How many alcoholic beverages does the majority of all female students in your department usually drink per drinking occasion? One alcoholic beverage (standard drink) is a small bottle of beer (0,33l), a small glass of wine or sparkling wine (0,125l) or a double schnapps (4cl)”]. The items were adopted from AUDIT-C for estimation of peers' alcohol frequency and quantity. We then multiplied frequency and quantity and thus obtained the frequency-quantity index for the individually estimated group norm.

### Personalized Normative Feedback

In 2016, we started the project ISPI (“Internet, Studierende, Peers & Intervention”) in cooperation with the Leibniz-Institut für Präventionsforschung und Epidemiologie in Bremen and implemented a first intervention. This 2016 intervention resulted in a change of difference to norms but not a change of behavior regarding the alcohol consumption of University students (57).

With the current intervention, we tried to strengthen and expand the effects of 2016. We further personalized the reference group for the University students by not only specifying it to their gender but also to their study department. With an even smaller comparison group we wanted to reach a higher degree of identification and a stronger effect of the intervention on both group norms and behavior. Additionally, we revised and clarified the normative feedback. We used PNF in the form of e-mails to reach as many University students as possible. One crucial advantage of e-mail-based interventions is that participants can access the information at any time or place whilst also protecting their anonymity (58). SNI with PNF represents a stand-alone, e-mail-based, personalized, normative feedback intervention.

The University students received feedback concerning their own as well as their peers' alcohol intake with regard to frequency and quantity as well as binge drinking behavior (defined as six or more alcoholic drinks per drinking occasion). The feedback consisted of three parts. In the first part, the University students received feedback of what they had indicated with regard to their own alcohol consumption (e.g., “You stated that you consume alcohol 3 to 4 times per week, usually 1 alcoholic drink per drinking occasion.” / “You stated that you drink more than 6 alcoholic drinks per drinking occasion once a month.”). In the second part, the University students received feedback with regard to their estimated alcohol intake of their peers i.e., the (mis-)perceived group norm (e.g., “You suppose, that the majority of the female students in your department consume alcohol 3–4 times per week, usually 2 alcoholic drinks per drinking occasion.”). In the third part, the students received feedback about their peers' alcohol intake (behavioral norm e.g., “In fact, the majority of the female students in your department consume alcohol 1–2 times per week, usually 2 alcoholic drinks per drinking occasion.”). Lastly, the University students received a statement indicating whether their alcohol intake was similar or above that of their peers.

### Data Analysis

We applied analyses of covariance (ANCOVA) to assess intervention effects with regard to the correction of overestimated group norms as well as University students' drinking behavior. We considered age, sex, self-efficacy and depressive symptoms as covariates. To evaluate the correction of overestimated group norms, we first calculated a frequency-quantity-index for the estimated as well as behavioral norms. Subsequently, we calculated a difference value between the individually estimated group norm (perceived alcohol intake of peers) and the behavioral norm (median, department- and gender-specific). With these indicators, we analyzed the difference values of both occasions for the intervention and control group. Finally, we assessed the changes in intervention and control group drinking behavior by comparing the AUDIT-C scores prior to and after the intervention. We used a 0.05 significance level.

## Results

We first identified the gender- and subject-specific behavioral norms for drinking frequency and drinking quantity that were also part of the intervention (feedback). Due to the high number of different behavioral norms that we computed for every gender and subject combination we only constitute the range of behavioral norms for the several combinations: Behavioral norms for quantity varied from 2 drinks to 4.5 drinks and behavioral norms for frequency varied from 1 time per month to 1–2 times per week.

With regard to hypothesis 1—the correction of overestimated individually estimated drinking norms—we observed a significantly larger reduction in the difference value (between the individually estimated group norm and the behavioral norm) in the intervention group compared to the control group [*F*_(2)_ = 8.46, *p* < 0.001, $\eta_{\text{p}}^{2}$ = 0.06]. This confirms hypothesis 1, namely that the intervention would correct misperceived individually estimated group norms in the intervention group.

With regard to hypothesis 2—the reduction of alcohol intake—we observed a significant reduction in their AUDIT-C scores [*F*_(2)_ = 3.96, *p* = 0.020; $\eta_{\text{p}}^{2}$ = 0.03]. Hence, the behavioral outcomes were in line with our hypothesis 2. Corrected means, standard deviations and test statistics of the outcome measures are depicted in Table 1.

**Table 1:** Corrected Means and test statistics of the ANCOVA outcome measures.

	**IG**	**CG**	**IG**	**CG**				
	**T1**		**T2**					
	**M (SE)**	**M (SE)**	**M (SE)**	**M (SE)**	**F ratio**	***df***	***p***	**η_p_^2^**
Difference value (individually estimated group norm – behavioral norm)	17.85 (1.32)	18.73 (1.77)	7.828 (1.00)	17.41 (1.34)	8.46^***^	2	<0.001	0.06
AUDIT-C score	4.92 (1.58)	5.20 (1.74)	4.31 (0.12)	4.94 (0.16)	3.96^*^	2	0.020	0.03

*Corrected means for the AUDIT-C score (sum of the three AUDIT-C items) and the difference value between individually estimated group norm (as a frequency-quantity index of the estimation) and the behavioral norm (also as a frequency-quantity index). Covariates (age, sex, self-efficacy, and depressive symptoms) were considered. IG, Intervention group; CG, Control group*;

* *p < 0.05*;

*** *p < 0.001*.

*T1: N_IG_ = 95 and N_CG_ = 179; T2: N_IG_ = 92, and N_CG_ = 180*.

## Discussion

We investigated whether a personalized, gender-specific social norms intervention for University students would affect their perception of their peers' alcohol intake as well as their own drinking behavior. Specifically, we compared the effects in the intervention group with a control group. Since most studies on SNI in University students were conducted in the USA, Australia, Brazil, New Zealand, Sweden, or the United Kingdom (41), our intervention constitutes one of the first studies evaluating SNI in German University students.

Our results are in line with our hypotheses. In contrast to the control group, participants in the intervention group ended up with a more realistic perception of their peers' alcohol intake. However, their perception was still above the group norm. Furthermore, participants of the intervention group reported a significantly larger reduction in the AUDIT-C score, which means that—compared to the control group—they drank less and less often after the intervention. However, their drinking level was still high yet lowered.

Our results confirm prior SNI research that found that University students reported significant decreases of their alcohol intake [e.g., (59, 60)]. There has, however, been serious criticism concerning SNI use with European populations. John and Alwyn (61) argue that there are important differences in campus life and in the definitions of alcohol misuse or heavy drinking between the UK and the US. They consider SNIs to be an ineffective tool in tackling heavy drinking behavior in European populations. Contrastingly, our study was able to validate SNI's efficacy in a European population and thus makes a valuable contribution to the knowledge about SNI.

We decided to use PNF in the form of e-mails to reach as many University students as possible. Since we integrated the intervention into our regular health monitoring survey, we showed that it is possible to implement both, health assessment and intervention simultaneously. This is a very effective and cost-efficient method. Still, there are several other feedback delivery options, in particular web/computer feedback, individual face-to-face feedback, group face-to-face feedback and general social norms marketing campaigns. In some cases, like when the intervention targets specific and small courses, it may be more appropriate to choose another delivery method, e.g., face-to-face feedback. Overall though, (e-)mailed feedback has been identified as one of the best delivery options for SNI (41).

The feedback in our study was department- and gender-specific. We do not know whether the overestimation of the group norms was affected by this choice of reference group. Galesic et al. (23) propose that the overestimation of people's behavior results from judging the behavior of a rather unfamiliar sample. Consequently, assessing the behavior of acquainted others might yield more realistic estimations. However, Giese et al. (25) have disproven this hypothesis. They showed that overestimation still occurs even when the reference group comprises only familiar people.

Not much research exists concerning the content and precise wording of the feedback. When studying the efficacy of a campaign to correct social group norm, Thombs et al. (62) found that only 38.5% of their sample understood the intended purpose of the campaign and its intervention. Therefore, we decided to not only include the participant's own and their peers' alcohol intake in the feedback but to also explicitly state whether the participant's alcohol intake was similar or above that of their peers. We also added whether or not their consumption would be categorized as problematic. We hoped that this information would made the feedback's intention easier to understand.

Furthermore, it is still rather unclear why SNI are more effective for some University students than for others. Giese et al. (25) have shown that University students with high self-control make more realistic estimations of their peers' alcohol consumption. There may be several other individual characteristics that impact the efficacy of SNI. We need much more knowledge on why University students overestimate peers' alcohol intake, and which University students are most vulnerable to such an overestimation, in order to target SNI most effectively. Other SNI studies suggest that several other contextual factors may influence its efficacy, e.g., social and environmental factors [availability of alcohol, acceptance of alcohol consumption in public; (63)]. Future research could also operationalize and control these social and environmental factors.

### Limitations

Our intervention is not free of shortcomings.

First, we used the AUDIT-C as an efficient, reliable and valid measure to assess the alcohol intake of the participants as well as the alcohol intake of their peers. The AUDIT-C has been successfully applied in previous SNIs (41). However, more direct behavioral measures such as the Alcohol Timeline Followback [TLFB; (64)] might be better suited to examining alcohol consumption and thus the effects of the intervention.

Second, a larger sample size might have improved and expanded our results. Since we included the intervention in our regular health monitoring survey, only 190 University students met our inclusion criteria for the intervention that we formulated a-priori (University students who overestimated their peers' alcohol use and indicated above average own consumption compared to peers' median alcohol use). Most of the studies on SNI targeted University students with increased risk (41), however, it may also be important to consider the intervention as a prevention tool for those who are not (yet) at increased risk or even at low-risk University students [e.g., (65)]. Furthermore, almost 75% of the participants were female, which also limits the generalizability of our results.

Third, we promised all interested University students who participated in our health monitoring survey a detailed feedback on their and their peers' alcohol intake, regardless of whether their intake was above the group norm. Hence, we did not randomly assign survey participants to either intervention or control group. A randomized assignment did not seem ethically justifiable as this would mean withholding the intervention from the control group or at least postponing their intervention. However, this selection procedure involves several shortcomings, especially the limited comparability between intervention and control group. Thus, we cannot rule out potential selection biases as would be possible with randomized control trials (RCT). RCTs randomly assign participants to either intervention or control group. Thus, RCTs are more comparative, minimize several biases (e.g., allocation or selection bias) and also minimize confounding factors. Since RCTs are the gold standard in interventional research, future studies should preferably use this design. Nevertheless, our design allowed us to control for known confounders and we therefore used sex, age, self-efficacy, and depressive symptoms as covariates within the ANCOVAs.

Fourth, although we asked participants if they received the intervention, we cannot be sure whether all of them read their feedback carefully and attentively. As described above, feedback of earlier studies was sometimes not clear enough, so we tried to keep the feedback as easy and understandable as possible. We are certain that the majority of University students was able to interpret it correctly.

Fifth, we used the second survey of our health monitoring to capture the effects of the intervention. This second survey was 12–16 weeks after the intervention. Unfortunately, we were not able to evaluate any long-term effects (e.g., after 1 year).

Sixth, we were able to observe significant differences in difference to norm (individually estimated group norm – group norm), AUDIT-C score. These effects were rather small in terms of effect sizes. However, in line with the prevention paradox, even small effect sizes can make a difference in such interventions.

### Conclusion

Our study proves SNI's overall efficacy in both norm and behavioral outcome variables. It is one of the first studies applying SNI in a German student sample. Since we focused on University students with overestimation of the group norm and an above average alcohol intake, we examined SNI's effect not only on University students with harmful alcohol consumption. Our intervention successfully addressed alcohol intake in University students with above average alcohol use. Therefore, SNI can also be used as a primary preventive instrument reducing alcohol use not only in those with problematic alcohol use.

Our study furthermore shows that it is possible to integrate SNI into regular health monitoring. This is an effective, cost-efficient, and pragmatic way to combine both, screening and intervention of alcohol misuse in University students. Along with environmental interventions and possible restrictions of alcohol promotion, SNI may be one important piece in the prevention of health problems due to alcohol misuse (59).

## Data Availability Statement

The raw data supporting the conclusions of this article will be made available by the authors, without undue reservation.

## Ethics Statement

The studies involving human participants were reviewed and approved by Ethics committee Freie Universität Berlin; FB Erziehungswissenschaft & Psychologie. The patients/participants provided their written informed consent to participate in this study.

## Author Contributions

CW, TL, and BG: conceptualization, investigation, validation, data curation, and project administration. CW, TL, BG, and TT: methodology. CW: formal analysis. CW, TL, BG, and A-CH: writing—original draft preparation. TL, CW, BG, A-CH, and TT: writing—review and editing. TL and CW: visualization. BG: supervision. All authors contributed to the article and approved the submitted version.

## Conflict of Interest

The authors declare that the research was conducted in the absence of any commercial or financial relationships that could be construed as a potential conflict of interest.
